# Supplementary material for: sORFdb – a database for sORFs, small proteins, and small protein families in bacteria
Source: BMC Genomics. 2025 Feb 5;26:110. doi: 10.1186/s12864-025-11301-w (PMC11796252; doi:10.1186/s12864-025-11301-w)
Supplement: Supplementary file 1 — Supplementary Material 1. [file 12864_2025_11301_MOESM1_ESM.pdf]

sORFdb – A database for sORFs, small proteins, and small protein families in bacteria

Author names:

Julian M. Hahnfeld[1*], Oliver Schwengers[1], Lukas Jelonek[1], Sonja Diedrich[1], Franz Cemič[2], Alexander Goesmann[1]

Affiliation:

[1] Bioinformatics and Systems Biology, Justus Liebig University Giessen, Giessen, Germany

[2] Department of Computer Science, University of Applied Sciences Giessen, Giessen, Germany

*** Corresponding author:**

E-mail: julian.hahnfeld@computational.bio.uni-giessen.de (JH)

Keywords:

small proteins, protein families, short open reading frames, sORF, database, bacteria

URLs:

GitHub: https://github.com/ag-computational-bio/sorfdb

Zenodo: DOI 10.5281/zenodo.10688271

Web: https://sorfdb.computational.bio/

Supplemental Tables

Table S1: Versions of the tools used for the download of the databases

**Tool**	**Version**
bioawk	1.0
curl	8.2.1
diamond	2.1.8
gawk	5.1.0
grep	3.11
hmmer	3.3.2
lxml	4.9.3
pigz	2.6
python	3.11.4
rename	1.601
tar	1.34
wget	1.20.3
xopen	1.7.0

Table S2: Versions of the tools used for the processing of the databases

**Tool**	**Version**
biopython	1.81
diamond	2.1.8
ijson	3.2.3
jq	1.6
krona	2.8.1
peptides	0.3.1
polars	0.16.14*
pyhmmer	0.9.0
pyrodigal	2.1.0
python	3.10.12
xopen	1.7.0

*The python script taxonomy2krona.py was executed with version 0.19.0.

Table S3: Versions of the tools used for the clustering

Tool	Version
blast	2.14.1
mcl	22.282
muscle	5.1
numpy	1.25.2
pandas	2.0.3
polars	0.18.15
pyhmmer	0.10.2
python	3.10.12
xopen	1.7.0

Table S4: Top 20 non-redundant small protein product annotations without ribosomal proteins

Product	Count
helix-turn-helix domain-containing protein	86298
helix-turn-helix transcriptional regulator	73168
transcriptional regulator	66725
acyl carrier protein	66706
antitoxin	61763
transposase	51557
type ii toxin-antitoxin system rele/pare family toxin	41659
dna-binding protein	35427
cold-shock protein	33735
sec-independent protein translocase protein tata	31886
xre family transcriptional regulator	28640
ferredoxin	26492
acylphosphatase	26294
hu family dna-binding protein	26157
type ii toxin-antitoxin system hica family toxin	25448
translation initiation factor if-1	25091
co-chaperonin groes	24978
exodeoxyribonuclease 7 small subunit	24499
exodeoxyribonuclease vii small subunit	24455
type ii toxin-antitoxin system phd/yefm family antitoxin	24142

Supplemental Figures

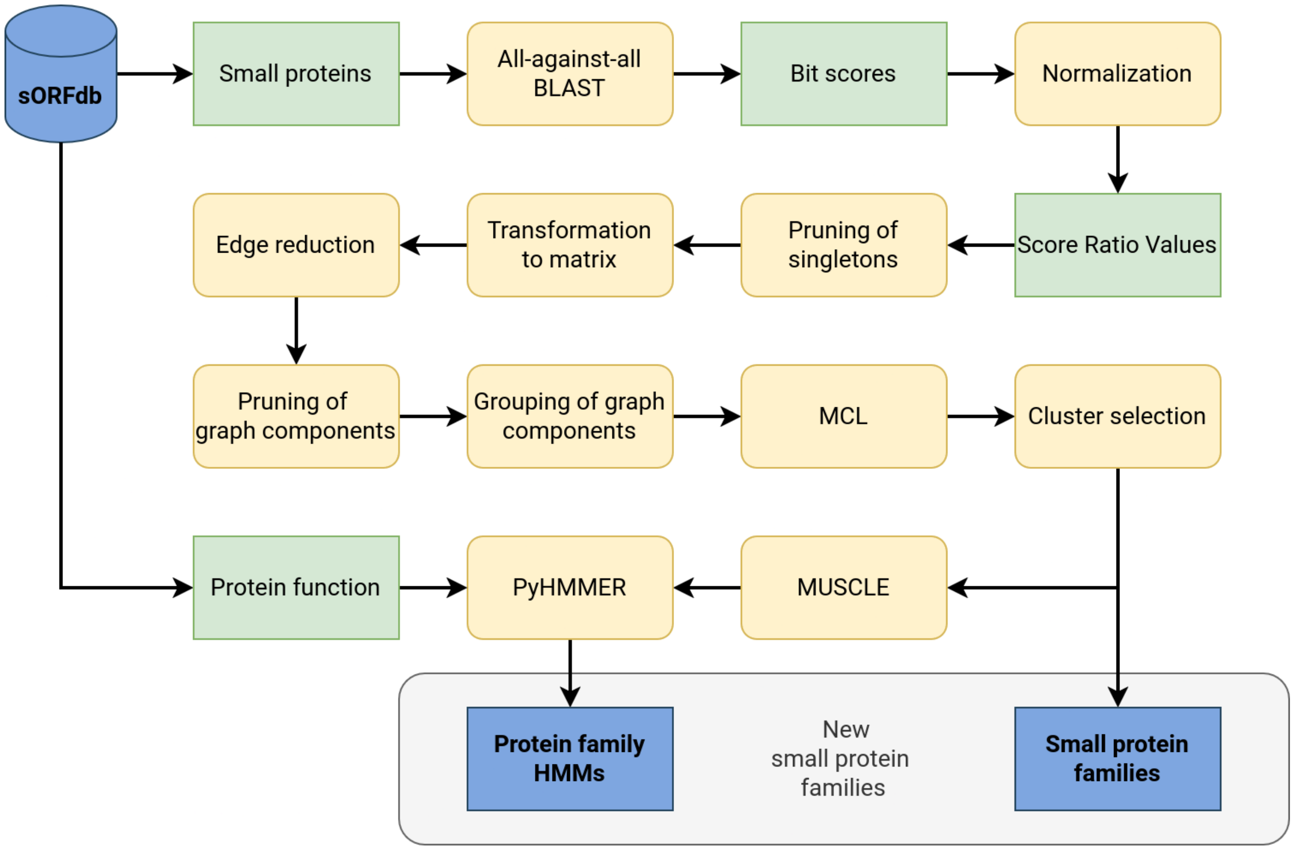

Figure S1: Scheme of the custom graph-based clustering approach
An all-against-all BLAST search was performed on the non-redundant small proteins in sORFdb. BLAST bit scores were normalized to SRVs and a lower threshold of 0.3 was used. Singletons were filtered out. The SRVs were transformed into a matrix representing the graph. To reduce high node degrees, only the *k* best edges of a node were kept without creating singletons. As an additional pruning step the heuristic proposed by Apeltsin et al. [40] was applied. Similar graph components were grouped into batches and Markov clustering was performed with different inflation values. Based on the highest efficiency score, the corresponding clustering for each batch was selected as the basis for the protein families. For families with at least five members, a multiple sequence alignment was computed with MUSCLE and HMMs were built with PyHMMER, for which functional descriptions were derived from the sORFdb entries.

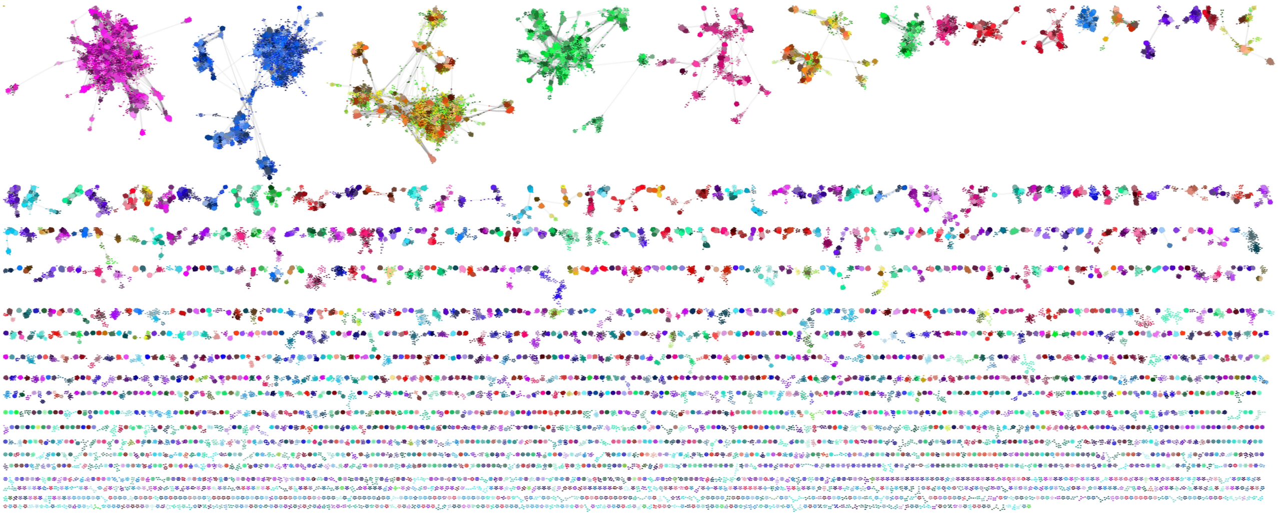

Figure S2: Visualization of the graph network of identified small protein families

All unconnected components of the graph were assigned a color palette. For the small protein families, one color is assigned in each component, where colors can occur multiple times. Families of one color separated by families of different colors are independent from another.
